# Structure-Based Design and Mechanistic Insight for Enhanced Catalytic Activity of Aldo/Keto Reductase AKR13B3 from *Devosia* A6-243 Toward T-2 Toxin

**DOI:** 10.3390/toxins18040158

**Published:** 2026-03-26

**Authors:** Jiali Liu, Huibing Chi, Xiaoyu Zhu, Qingwei Jiang, Zhaoxin Lu, Ping Zhu, Fengxia Lu

**Affiliations:** College of Food Science and Technology, Nanjing Agricultural University, Nanjing 210095, China

**Keywords:** T-2 toxin, 3-keto-DON, AKR13B3, catalytic activity, 3-D structure-based design, molecular mechanism

## Abstract

Trichothecene mycotoxins, especially T-2 toxin, represent a significant threat to food safety and public health. Although the enzymatic degradation of deoxynivalenol has been extensively investigated, there are few reports of enzymes capable of efficiently degrading T-2 toxin. This study identified that the aldo-keto reductase AKR13B3 from *Devosia* A6-243 exhibits 3-keto-DON-degrading and a little T-2 toxin-degrading activity. To address this limitation, a rational design strategy targeting the substrate-binding pocket was employed to enhance its activity. Utilizing site-directed and combinatorial mutagenesis, a double mutant R134F/D217A was successfully screened. R134F/D217A retains catalytic activity towards 3-keto-DON while significantly enhancing its catalytic capacity for T-2. Specifically, the R134F/D217A variant exhibited a 2.88-fold increase in catalytic activity and a 3.15-fold enhancement in catalytic efficiency (*k_cat_*/*K_m_*) relative to the wild type enzyme. Notably, a substantial improvement in thermal stability was also observed. After incubation at 55 °C, the residual activity of the R134F/D217A mutant was 2.63 times that of the wild type. Molecular dynamics (MD) simulations and three-dimensional structural modeling suggested the mechanistic basis for the enhanced performance of the R134F/D217A double mutant. Catalytic enhancement stems from a shortened nucleophilic attack distance, a positively biased electrostatic environment, combined with an enlarged pocket and reduced binding free energy. Concurrently, the increased thermal stability results from decreased flexibility and a more rigid structural architecture. This work presents the first report of AKR13B3 as an effective enzyme for T-2 toxin transformation, and its catalytic activity was significantly enhanced through rational design. Thus, a novel enzymatic strategy was proposed, and could inform future approaches to study issues related to T-2 toxin contamination.

## 1. Introduction

Mycotoxin contamination poses a serious global threat to human and animal health. Among the most toxic mycotoxins are Trichothecenes, primarily produced by the genus *Fusarium* [1]. These compounds significantly compromise food safety due to their severe toxicological effects, including immunosuppression, gastrointestinal damage, and carcinogenicity. Trichothecenes are characterized by the tetracyclic sesquiterpenoid 12,13-epoxytrichothec-9-ene ring [2,3]. T-2 toxin, a type A trichothecene, is particularly notable for its acute toxicity. Its molecular structure, characterized by epoxy groups, a double bond, and an ester side chain at the C12 and C13 positions (Figure S1), is responsible for its potent harmful effects [2]. Upon ingestion, T-2 toxin primarily targets the digestive tract, causing extensive damage to the mucosal lining, which can lead to hemorrhagic gastroenteritis and, frequently, death within 24–72 h [1,4,5]. Therefore, developing effective strategies to degrade T-2 toxin in contaminated food and feed is urgently needed.

Currently, effective strategies for T-2 toxin detoxification remain remarkably scarce. Traditional physical and chemical detoxification approaches are often costly, may leave harmful residues on food and feed surfaces, and generally exhibit inadequate detoxification efficacy [6]. By contrast, biological detoxification methods are distinguished by their remarkable advantages of environmental benignity, high safety, and superior efficiency [7], holding considerable promise for applications in food processing and safety [8,9]. While certain bacterial strains, such as *Rhodococcus erythropolis* NI1, have been shown to degrade T-2 toxin, the resulting products may retain toxicity, and the microbes themselves can be difficult to isolate or are potentially hazardous [10]. Given their safety profile and catalytic efficiency, enzymes capable of degrading T-2 toxin have attracted growing interest in industrial and environmental biotechnology [11]. A leading candidate is the aldo-keto reductase AKR13B3 [12], sourced from *Devosia* bacteria. This enzyme efficiently degrades the mycotoxin deoxynivalenol (DON) and its metabolite 3-keto-DON in an NADPH-dependent manner, achieving up to 100% substrate conversion under assay conditions [3,13]. Nevertheless, its activity toward the structurally distinct T-2 toxin has not been established, and no attempts to engineer this enzyme for T-2 toxin degradation have been reported to date. 

To bridge this gap, protein engineering methods offers powerful tools to enhance enzyme function [14]. These techniques primarily encompass irrational design, semi-rational design, and 3D structure-based rational design [15,16]. Both of the first two strategies depend on the establishment of high-throughput screening methods requiring considerable labor and offering limited screening efficiency [17], which enables the construction of a small yet precise library of mutants based on the relationship between enzyme structure and function, thereby hopefully achieving efficient enzyme engineering [18]. For instance, He et al. [19] analyzed the structure of the complex formed by AKR13B2 and NADPH and identified its catalytic triad (D63, Y84, K117) and substrate-binding pocket; these are all closely related to the enhancement of catalytic activity. Correspondingly, Luo et al. [20] performed saturation mutagenesis on the aldo-keto reductase KIAKR (EC 1.1.1.26) and identified the mutant Y259W/W296L, which showed a 10.25-fold increase in catalytic efficiency, but only for the substrate t-butyl 6-cyano-(5R)-hydroxy-3-oxohexanoate. However, the rational design of enzymes specifically aimed at T-2 toxin detoxification remains largely unexplored [21].

This study characterized the T-2 toxin catalytic activity of the aldo-keto reductase AKR13B3 from *Devosia* sp., filling a gap in the limited body of research on the enzymatic transformation of T-2 toxin. To address its initially low catalytic activity, we employed a rational design strategy targeting the substrate-binding pocket, thereby engineering a variant with significantly improved catalytic efficiency. Furthermore, molecular dynamics simulations and structural modeling provided insights into the molecular mechanism underlying the enhanced properties of the AKR13B3 mutant. These findings demonstrate that AKR13B3 can be engineered to enhance its T-2 toxin transformation activity, providing a basis for further exploration of enzymatic approaches to study T-2 toxin contamination.

## 2. Results

### 2.1. Selection of the Target Residues for Improving Catalytic Activity of Wild Type

**Figure 1:**
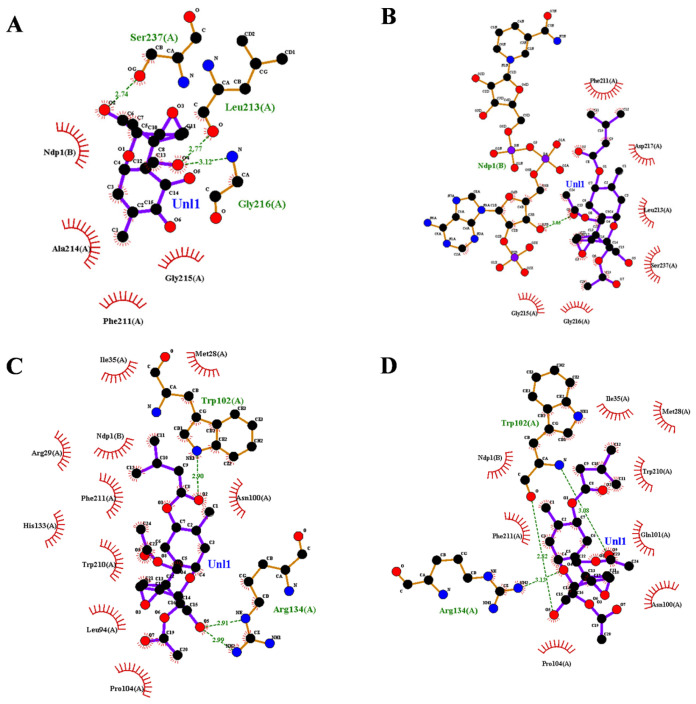
Interaction diagrams of wild-type AKR13B3 with 3-keto-DON and T-2 toxin. (**A**) Hydrogen bonds and hydrophobic interactions between the wild-type protein and 3-keto-DON. (**B**–**D**) Three representative binding conformations of the wild-type protein with T-2 toxin obtained from molecular docking. All interaction analyses were performed using LigPlot+ software v.2.2.9 [25].

The three-dimensional structure of AKR13B3 in complex with its cofactor NADPH was initially predicted using AlphaFold 3 [22,23], and the highest-scoring model was selected for subsequent analyses (Figure S2). Following this, semi-flexible docking of the substrates 3-keto-DON and T-2 toxin into the AKR13B3 structure was performed using AutoDock Tools 1.2.3 [24]. The optimal docking poses for each substrate were compared to identify key amino acid residues within the binding pocket (Figure S3). To prioritize mutations that would not compromise the enzyme’s activity toward 3-keto-DON, residues forming hydrogen bonds with 3-keto-DON (Ser237, Leu213, Gly216) and those involved in hydrophobic interactions with both substrates (Gly215, Phe211) were excluded from mutagenesis. This analysis identified Ala214 and Asp217 as key residues for engineering (Figure 1A,B). Examination of two other high-ranking docking models further revealed that Trp102 and Arg134 interacted with T-2 toxin via hydrogen bonds but showed no interaction with 3-keto-DON, leading to their selection as additional key residues (Figure 1C,D).

### 2.2. Impact of Key Residues on Catalytic Activity Differences in Wild Type

**Figure 2:**
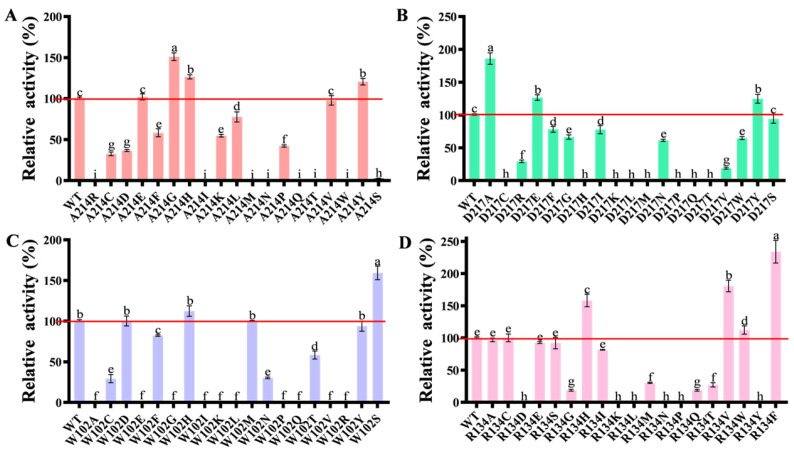
Relative activity of mutants after site-directed mutagenesis; (**A**) 214-site, (**B**) D217-site, (**C**) W102-site, (**D**) R134-site. Note: different lowercase letters in histogram indicate that there is significant difference at 0.05 level. The same applies to the following.

**Figure 3:**
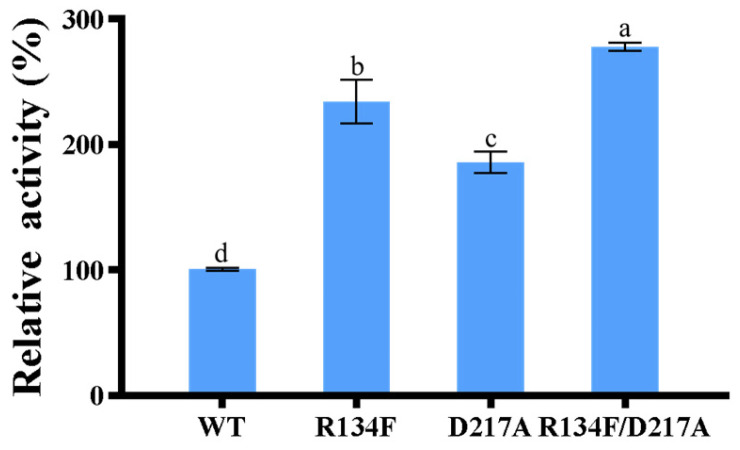
Relative activity of double-point combination mutation. Note: different lowercase letters in histogram indicate that there is significant difference at 0.05 level. The same applies to the following.

In the preliminary work of our laboratory research group, it has been confirmed that AKR13B3 can degrade 3-keto-DON, and that the degradation product is 3-epi-DON. The reaction site is a C3 ketone group [12]. In the present study, as shown in Figure S6A,B, T-2 toxin exhibited a retention time of approximately 6.9 min. Comparison of the control sample (Figure S6A) with the mutant enzyme-treated sample (Figure S6B) revealed that both samples displayed a peak at around 6.9 min, and the peak area of the control sample (Figure S6C) was significantly larger than that of the treated sample (Figure S6D). This result suggests a substantial decrease in T-2 toxin content after 24 h of enzymatic reaction. Furthermore, a new chromatographic peak appeared at approximately 9.0 min in the mutant-treated sample (Figure S6F), which was absent in the control (Figure S6E). LC–MS analysis suggested that this peak corresponds to a compound with a molecular formula of C24H32O12, tentatively assigned as a potential T-2 toxin transformation product. The mass spectrometric features are consistent with redox modifications at the C3′ and C4′ positions [26]. To enhance the likelihood of success, each of the four identified key residues was individually mutated to 19 other amino acids. All mutants were successfully expressed in *E. coli* with protein yield and purity comparable to the wild type AKR13B3, indicating equally efficient enzyme expression (Figure S4). Activity assays revealed that none of the mutations at these four positions significantly affected catalytic activity toward 3-keto-DON (Figure S5), while the activity against T-2 toxin was enhanced to varying degrees. For position Ala214, mutants A214G, A214H, and A214Y showed activities 1.5-fold, 1.26-fold, and 1.2-fold higher than the wild type, respectively (Figure 2A). At Asp217, mutants D217A, D217E, and D217Y exhibited 1.86-fold, 1.27-fold, and 1.25-fold increases in activity. The most notable improvement at Arg134 was observed with the R134F mutant, which showed a 2.34-fold enhancement in activity (Figure 2C). For Trp102, only the W102S mutant conferred a significant activity increase, achieving 1.6-fold of the wild-type activity (Figure 2D). These results confirmed these positions as critical hotspots influencing catalytic activity. To further boost T-2 toxin degradation, the two most effective single mutants, R134F and D217A, were combined into a double mutant. Screening showed that the catalytic activity of the double mutant R134F/D217A was 2.88-fold that of the wild type, outperforming the R134F and D217A single mutants by 12.3% and 55%, respectively (Figure 3, Table S2).

### 2.3. Enzymatic Characterization of Wild Type and Its Mutants

**Figure 4:**
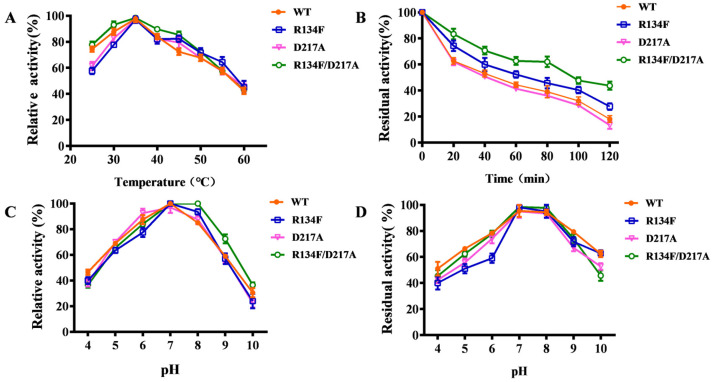
Enzymatic properties of wild type and its mutants; (**A**) optimum temperature range for the activity of AKR13B3 and its mutants; (**B**) thermostability of AKR13B3 and its mutants at 55 °C; (**C**) optimal pH for AKR13B3 and its mutants; (**D**) pH stability of the wild type and mutants.

To investigate the enzymatic properties of AKR13B3 and its mutants, T-2 toxin was used as the substrate. The optimum reaction temperature was first determined within a range of 25–60 °C. Both the wild-type and mutant enzymes exhibited the same optimum temperature of 35 °C (Figure 4A). After incubation at 55 °C for 100 min, wild-type AKR13B3 retained only 40% of its initial activity. In contrast, the R134F/D217A and R134F mutants maintained over 40% residual activity under the same conditions. Among all tested variants, the double mutant R134F/D217A shows the highest thermostability, retaining more than 45% of its activity even after 120 min at 55 °C (Figure 4B), indicating a simultaneous enhancement in both catalytic activity and thermal stability.

The optimum pH range was identified as pH 4.0–10.0. As shown in Figure 4C, the wild-type, R134F, and D217A enzymes all displayed maximum activity at pH 7.0, whereas the optimum pH for R134F/D217A shifted to 8.0. The relative activity of all enzymes remained above 50% within the pH range of 7.0 to 9.0. However, the R134F, D217A and R134F/D217A mutants exhibited similar stability to the wild type within the pH range of 7.0–8.0, and there was no significant improvement in pH stability.

Kinetic parameters further revealed changes in catalytic performance (Table 1). The *K_m_* values of the mutant enzymes R134F, D217A, and R134F/D217A were 211.56 ± 8.12, 247.58 ± 4.59, and 163.61 ± 7.12μM, respectively, corresponding to 74%, 86%, and 57% of the *K_m_* value of WT (286.30 ± 15.01μM). This reduction relative to the WT indicates that the mutants exhibit enhanced substrate affinity, thereby facilitating substrate access to the active site. The catalytic efficiency (*k_cat_*/*K_m_*) of the wild-type and R134F/D217A mutant enzymes were determined to be 2.31 mM^−1^·s^−1^ and 7.27 mM^−1^·s^−1^, respectively. This difference represents that the mutant exhibits a catalytic efficiency 3.15 times that of the wild type. In summary, the R134F/D217A mutant exhibits superior catalytic properties.

### 2.4. Molecular Dynamic Analysis of Wild Type and Its Mutant

**Figure 5:**
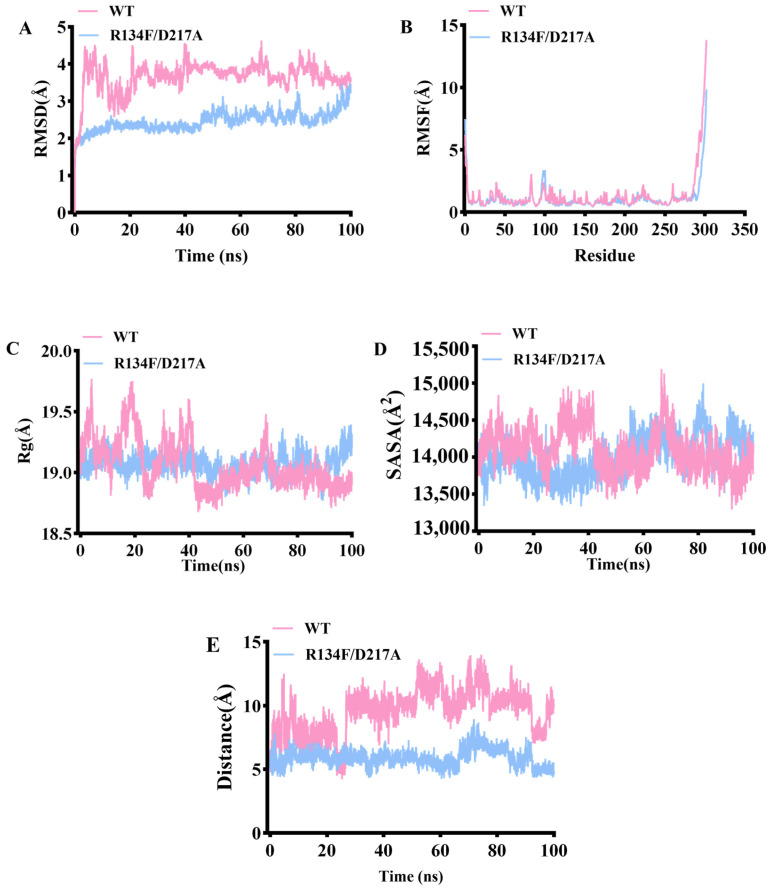
RMSD (**A**), RMSF (**B**), Rg (**C**), SASA (**D**) and nucleophile-attack distance plot (**E**) values of wild type and mutant R134F/D217A at 298.15 K.

To explore the mechanism underlying the improved catalytic activity and thermostability of the R134F/D217A mutant, molecular dynamics (MD) simulations of the wild-type and mutant enzymes were performed at 298.15 K for 100 ns. Root mean square deviation (RMSD) serves as a crucial parameter for evaluating fluctuations in catalytic stability and thermal stability within protein conformations [27]. As shown in Figure 5A, the RMSD of the WT complex rapidly increased within the first 10 ns and remained stable at approximately 3.5–4.0 Å, whereas the R134F/D217A complex maintained an overall RMSD of about 2.0–2.5 Å with smaller fluctuations. This suggests that the R134F/D217A complex tended to exhibit greater structural stability during the simulation, with a lower overall conformational deviation from the initial structure [28]. RMSF values reflect the fluctuation of amino acid residues in proteins [27]. As shown in Figure 5B, the fluctuation trends of residues in both complexes are largely consistent, with most regions exhibiting RMSF values below 2 Å. Only the C-terminal region shows a distinct fluctuation peak. Overall, the difference in residue flexibility between R134F/D217A and WT complexes is not significant, suggesting that the mutation did not substantially alter the local flexibility distribution of residues, with only a slight reduction in fluctuation observed in the terminal regions. The radius of gyration (Rg) is related to the structural stability of a protein, and smaller Rg values may indicate a more stable protein structure [27]. The Rg for the WT complex decreased slightly and exhibited some fluctuation during the simulation, while the R134F/D217A complex remained stable between 19.0 and 19.4 Å (Figure 5C). This suggests that the R134F/D217A complex has a more compact overall structure and a more stable folded state. Solvent accessible surface area (SASA) values represent the aggregation-related properties of protein and a smaller SASA value may imply a tighter packed structure of protein [27]. The R134F/D217A (Figure 5D) complex showed overall lower values with smaller fluctuations, which may be consistent with more stable surface exposure after the mutation. This may support that the R134F/D217A complex had a tighter and more stable structural conformation.

In addition, the nucleophilic attack distance between the C4 site of NADPH and the key toxic group at the substrate T-2 toxin is also a crucial factor that influences the catalytic activity of R134F/D217A [12,29]. In the WT system, the observed distance is highly dynamic (8–10 Å, peaking at 14 Å), suggesting significant instability in the reactive conformation. This dynamic instability may hinder the formation of a productive geometry necessary for efficient catalysis (Figure 5E). In contrast, the R134F/D217A system maintains an overall stable distance of approximately 5–6 Å, with markedly reduced fluctuations, and this distance approaches the geometric threshold required for efficient reactions. This observation suggested that R134F/D217A may significantly enhance the binding stability between the substrate and NADPH, which could potentially contribute to improved catalytic efficiency.

Based on trajectories derived from molecular dynamics simulations, binding energies were calculated utilizing the MM-GBSA method [30,31], which more accurately reflects the binding efficiency between T-2 toxin and the protein. As illustrated in Table 2, the binding energy between T-2 toxin and the WT complex was determined to be −16.57 ± 1.43 kcal/mol, whereas between T-2 toxin and the R134F/D217A complex was −21.31 ± 2.50 kcal/mol. Negative values indicate the presence of binding affinities between these molecules and the target protein, with more negative values signifying stronger binding interactions. The calculations suggest that the binding affinity between T-2 toxin and the double mutant is higher [32]. Energy decomposition analysis suggests that van der Waals forces constitute the primary contribution to their binding, followed by electrostatic interactions and nonpolar solvation free energy. Altogether, the stable protein structure may contribute to the enhanced catalytic activity of mutant R134F/D217A.

### 2.5. Three-Dimensional Structure Analysis of Wild Type and Its Mutant

**Figure 6:**
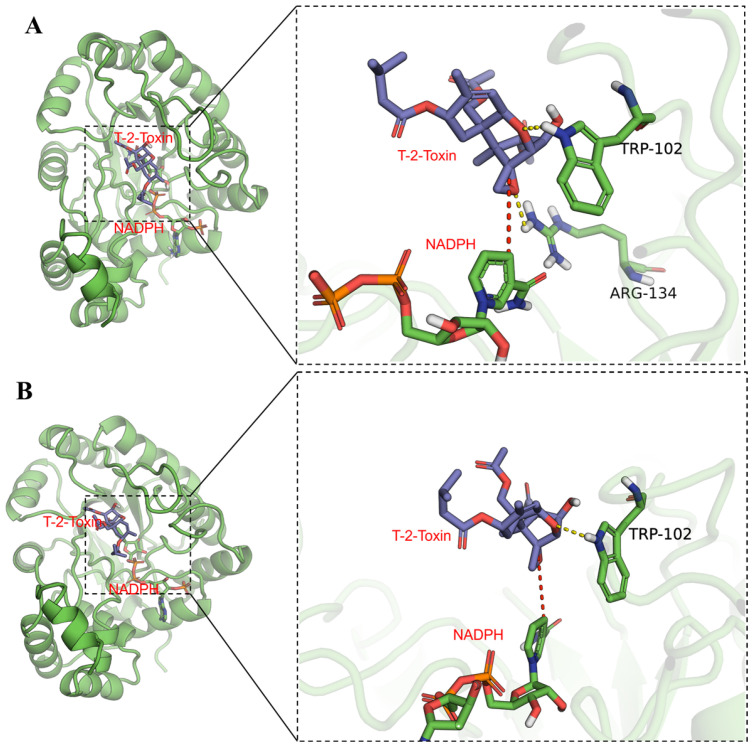
Docking-derived binding models of T-2 toxin and NADPH with wild type and the R134F/D217A mutant. (**A**) Based on the docking-derived binding model of WT/NADPH/T-2 toxin; (**B**) based on the docking-derived binding model of R134F/D217A/NADPH/T-2 toxin. The left image shows the overall view while the right image displays a local view. Blue sticks represent the small molecule, green sticks denote NADPH, green cartoons indicate the protein, yellow dashed lines indicate hydrogen bonds, and red dashed lines connect the reaction sites.

**Figure 7:**
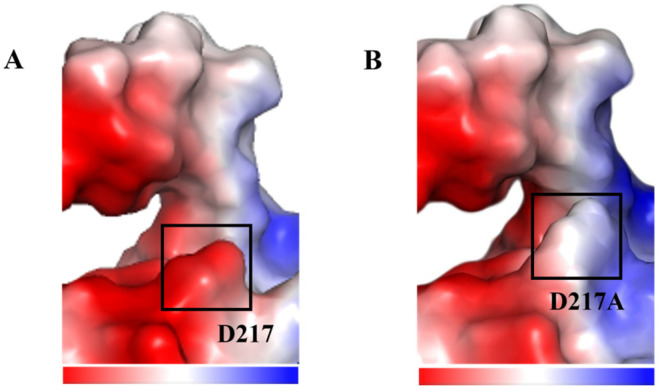
Surface electrostatic potential map of the wild type (**A**) and R134F/D217A mutant (**B**). Positive, negative, and neutral values of electrostatic potentials are represented by shades of blue, red, and white color respectively.

Furthermore, we sampled the binding conformation of wild type and mutant separately with T-2 toxin at 100 ns after simulations and analyzed their binding patterns. As illustrated in Figure 6, the enzymes and substrate were both well stacked. The mutated sites of R134F/D217A did not affect the relative positions of enzyme or substrate but slightly differed in detail; through observation of the binding modes exhibited by the WT and NADPH in complex with T-2 toxin, it was determined that T-2 toxin binds to a region on the protein adjacent to NADPH, with the respective reaction sites positioned in close proximity. Specifically, hydrogen bond interactions are formed between T-2 toxin and the protein residues W102 and R134, whereas R134 engages in interactions with the tricyclic heterocyclic ring. This interaction is postulated to confer stability upon the tricyclic heterocyclic ring, thereby potentially impeding structural rearrangement during the reaction process. The larger pocket facilitates substrate binding to the active site. Thus, mutant R134F/D217A easily hydrolyzed the substrate with an increased catalytic efficiency [33]. Notably, R134F/D217A exhibits two key changes that may enhance activity. First, the R134F mutation disrupts hydrogen bonds with T-2 toxin, which may facilitate the modification of its structure. Second, the double mutation increases flexibility near the active site, potentially allowing more substrates to enter the pocket and thus improving catalytic efficiency [34]. Furthermore, the mutation of residue D217 to D217A eliminates the negative charge near the active site (Figure 7), shifting the surface electrostatic potential from “locally negatively charged” to “neutral and slightly positive”. This change enhances electrostatic attraction with the partially negative oxygen of the T-2 toxin ester group, potentially facilitating substrate binding and stabilizing the negatively charged transition state [35], ultimately enhancing catalytic activity [36]. Thus, the enhanced catalytic activity of the R134F/D217A mutant may be attributed to the mutation at the R134 site eliminating hydrogen bond interactions with T-2 toxin and enlargement of the substrate pocket.

## 3. Discussion

T-2 toxin and 3-keto-DON (Figure S1) are both members of the trichothecene family of mycotoxins and share an identical tetracyclic core skeleton, which includes the 12,13-epoxide group. This epoxide moiety is a defining feature of trichothecene mycotoxins and is directly responsible for their potent toxicity. These toxins are widely present in grains and animal feed, posing a serious threat to food safety [37,38]. Traditional degradation approaches, including physical adsorption and chemical degradation, are associated with inherent limitations, such as incomplete toxin removal, destruction of nutritional components, and generation of secondary pollutants [6]. In contrast, the enzymatic degradation achieved by the inactivation of toxic active functional groups through hydrolysis exhibits high efficiency, environmental friendliness, and strong target specificity. As a result, such enzymatic degradation has become a core focus in current biodegradation research [39]. This study found that AKR13B3, known for its high efficiency in degrading 3-keto-DON, also possesses the ability to degrade T-2 toxin. However, the AKR13B3 enzyme suffers from limitations including low catalytic efficiency and weak substrate affinity, which hinder its large-scale production in industry. In recent years, rational design has emerged as a highly efficient strategy for modifying enzyme catalytic activity, leveraging its directed utilization of enzyme structure–function relationships [40]. Hence, improving the catalytic activity of AKR13B3 toward T-2 toxin through rational design appears to be a feasible and effective approach.

Based on the three-dimensional structure of AKR13B3 and molecular docking results, four “hotspot” amino acid residues were identified: R134, D217, W102, and A214 [27]. Significant enhancement of T-2 toxin-degrading catalytic activity was achieved via rational design strategies, including site-directed saturation mutagenesis and mutant combination. In initial experiments, six single-point mutants with substantial catalytic improvement were screened through T-2 toxin residual rate detection; these mutants were A214G, A214H, D217A, R134F, R134V, and W102S. To obtain mutants with higher catalytic efficiency, the two single-point mutants exhibiting the highest relative activity were subjected to combinatorial mutagenesis, resulting in the double-point mutant R134F/D217A; its catalytic activity is 2.88-fold that of the wild type. Subsequent enzymatic studies revealed that the R134F/D217A mutant possessed superior pH and thermal stability over the wild type, concurrent with a 3.15-fold increase in *k_cat_*/*K_m_*, which signifies a markedly enhanced catalytic efficiency. Theoretically, saturation mutagenesis at four sites (20 × 20 × 20 × 20) [41] would enable comprehensive exploration of sequence space. However, given current limitations in experimental throughput, this study adopted a focused rational design strategy targeting key functional residues. Based on structural analysis, four hotspot residues most likely to influence catalytic activity were prioritized for combinatorial optimization. This “small but precise” approach avoids generating large numbers of unstable or non-functional variants, thereby maintaining screening efficiency and data quality. Using this strategy, the R134F/D217A double mutant was successfully obtained, exhibiting significantly enhanced catalytic efficiency. It should be noted, however, that higher-order combinations of other beneficial single mutations may yield additional synergistic effects. Therefore, future work will systematically explore combinatorial mutations at more sites, employing machine learning-assisted directed evolution or high-throughput screening to construct libraries incorporating multiple beneficial mutations. The dataset generated here will also be combined with active learning models to progressively explore broader sequence space while controlling experimental costs. Such efforts will help elucidate epistatic interactions and may inform future strategies for the enzymatic transformation of T-2 toxin.

Subsequently, molecular dynamics simulations were performed on the engineered enzyme to explore the structural basis for its enhanced catalytic activity [42]. A similar approach was adopted by Niu et al. [12], who analyzed the attack distance between the C3 atom of DON and the C5 atom of PQQ. In that study, the most frequently observed distance in the wild-type DADH was 4.98 Å, which was 0.63 Å longer than that in the M5-1 variant (4.35 Å), suggesting that the reduced distance contributed to the improved catalytic efficiency of the mutant. This observation is consistent with the principle reported by Niu et al. [12] as the attack distance between the NADPH C4 atom and the T-2 C12 atom was 9.0 Å in the wild-type enzyme, compared to 5.5 Å in the R134F/D217A mutant. The reduced distance observed in the mutant suggests that a more favorable geometry for hydride transfer may contribute to its enhanced catalytic activity. Directly relevant to our study is the T-2 toxin-transforming enzyme derived from Fhb7, as reported by Yang et al [3]. Its catalytic mechanism involves key residues that facilitate the modification of T-2 toxin [3]. Zhou et al. [34] introduced point mutations that reduced hydrogen bonding and increased the flexibility of the loop region near the active site, thereby facilitating the entry of more substrates into the pocket and enhancing the catalytic activity of the mutant. Similarly, our study also validated the aforementioned findings by introducing the R134F mutation, which disrupts the hydrogen bond with T-2 toxin. This disruption likely facilitates substrate access to the pocket, ultimately leading to an enhanced catalytic activity. Wang et al. [43] calculated binding free energy changes before and after enzyme mutation, revealing that reduced enzyme–substrate binding free energy underlies the enhanced catalytic activity of mutant enzymes. Our study indicates that the absolute value of the total binding free energy for the mutant enzyme R134F/D217A is significantly higher than that of the wild type. This higher binding free energy favors the spontaneous formation of a stable complex between the enzyme and its substrate to enhance the catalytic activity of the enzyme [44]. Additionally, the study by Cao et al. [36] focused on non-catalytic region mutations (Cys69Tyr, Asn136Asp) in Class A β-lactamases. They found that although these mutations do not directly act on catalytic residues, they trigger a cascade of structural changes, ultimately altering the electrostatic distribution in the active site and enhancing catalytic efficiency. The R134F/D217A double mutant provides an example consistent with this hypothesis. Here, the D217A substitution neutralizes a negative charge in the active site vicinity, shifting the local electrostatic potential from negative to weakly positive. This altered electrostatic environment may enhance interactions with the partially negative carbonyl oxygen of the T-2 toxin ester group, offering a plausible structural rationale for the observed increase in catalytic activity. Consequently, this optimization not only promotes substrate binding but also stabilizes the anionic transition state of the proposed modification reaction, leading to a net enhancement in catalytic activity.

Molecular dynamics analysis revealed that the R134F/D217A mutant exhibited lower and more stable RMSD, Rg, and SASA values than the wild type, which may be consistent with enhanced structural rigidity, superior internal packing, and a stabilized hydrophobic core. This computationally observed increase in structural integrity is consistent with the mutant’s experimentally superior thermal stability at 55 °C, offering a potential structural explanation for its enhanced resistance to thermal denaturation. This finding strongly resonates with the work of Wang et al. on the glycosylation engineering of Rhizopus oryzae lipase (ROL) [45]. In their study, the introduction of N-glycosylation in the optimal mutant N227 resulted in a half-life of 298.8 h at 45 °C, which is 7.23 times longer than that of the parent strain, while its catalytic activity also increased by 29.2%. Molecular dynamics simulations revealed that the incorporated glycan enhances protein rigidity and forms strong hydrogen bonds with the protein, thereby stabilizing the overall structure of the lipase. This mechanism of thermal stability enhancement aligns well with the characteristics observed in our study for the R134F/D217A, namely lower RMSD, Rg, and SASA values, which collectively indicate enhanced structural rigidity, reduced conformational fluctuations, and a more stable hydrophobic core. It is important to note that the proposed mechanism is derived from molecular docking simulations and remains hypothetical, requiring experimental validation in future studies. An authoritative study developed T-2 toxin-degrading enzymes through ancestral sequence reconstruction, achieving simultaneous enhancement of thermostability and catalytic activity [3]. Similarly, the mutant R134F/D217A in this study shows synchronized improvement in both catalytic activity and thermal stability. This demonstrates that the engineered enzyme exhibits simultaneously enhanced catalytic activity and thermal stability, addressing a key limitation in T-2 toxin degradation research. Both studies employed molecular dynamics simulations to uncover the molecular mechanisms behind the enhanced catalytic activity and thermostability, albeit from complementary perspectives. The study by Yang et al. [3] utilized simulations to identify key local features, specifically residue F27 and a long insertion loop in mutant N2, which are critical for substrate recognition. In contrast, our simulations suggest that the improved performance may be attributed to enhanced global structural rigidity, reduced conformational fluctuations, and increased compactness.

Altogether, through rational design, we developed a superior T-2-transforming enzyme characterized by significantly boosted catalytic activity and thermal stability, while retaining its activity toward 3-keto-DON. Molecular dynamics simulations provided insights into the structural basis underlying its improved performance. This work presents an engineered biocatalyst with promising potential for the enzymatic transformation of trichothecene mycotoxins. It should be noted that the proposed structure of the transformation product (3′-OH-4′-COOH-T-2) is tentatively assigned based on LC–MS data and is consistent with previously reported transformation products in the literature. Further validation using high-resolution mass spectrometry and NMR analysis would help to confirm this structural assignment in future studies. The typical mechanism of aldehyde-ketone reductases involves a conserved four-nucleus catalytic structure that facilitates hydrogen transfer from the C4 position of NADPH nicotinamide to the substrate, consistent with the degradation pathway of 3-keto-DON by AKR13B3. Based on existing literature, we currently hypothesize that atom transfer may occur at the C3′ and C4′ positions of T-2 toxin. Therefore, further confirmation using HRMS/MS and NMR is required in future studies to validate the structural assignment. Additionally, AKR13B3 requires NADPH as an essential cofactor for catalytic activity. The high cost and instability of NADPH currently pose challenges for the practical application of NADPH-dependent enzymes. Hence, further efforts, such as the development of efficient cofactor regeneration systems or cofactor engineering to utilize more stable and cost-effective cofactors, are needed to facilitate practical applications.

## 4. Conclusions

In conclusion, we report the rational design of a T-2 toxin-transforming enzyme from a *Devosia* species. By targeting the binding pocket, we obtained a variant, R134F/D217A, with significantly enhanced catalytic activity and thermal stability, while its activity toward 3-keto-DON was preserved. This work highlights the potential of structure-guided engineering for improving mycotoxin-degrading enzymes and provides a valuable candidate for further development in bioremediation strategies. Molecular dynamics and structural simulations elucidated the mechanism behind the superior performance of the double mutant. This study has yielded a novel enzyme with high catalytic potency and remarkable thermal stability for degrading trichothecene mycotoxins, including T-2 toxin, suggesting its potential utility in biotransformation applications

## 5. Materials and Methods

### 5.1. Strains and Chemicals

The *E. coil* BL21 (DE3) strain (in our laboratory) was used as the host for protein expression (Figure S8) [12,46]. The plasmid pET-28a-akr13b3 was used as the expression vector. The substrate T-2 toxin was obtained from Pribolab (Qing Dao, China). Gel purification, recombination, plasmid extraction, and other biochemical regents test kits were obtained from vazyme (Nanjing, China). All relevant primers were synthesized by GenScript (Nanjing, China). Unless otherwise stated, all other chemicals and reagents used in this study were of analytical grade and commercially available.

### 5.2. Molecular Modeling and Structural Analysis

The protein and NADPH structures used for docking were predicted using AlphFold 3. The 3D structure of the small molecule T-2 toxin was obtained from PubChem and minimized under the MMFF94 force field. Molecular docking was performed using AutoDock Vina. Prior to docking, the predicted complex structure was optimized through kinetic simulations and the receptor protein was processed with PyMol 2.5.2 [47] to remove water molecules, salt ions, and the small molecule. Additionally, ADFRsuite 1.0 [24] was employed to convert all processed small molecules and the receptor protein into the PDBQT format required for AutoDock Vina docking [48]. During docking, the global search exhaustiveness was set to 32, with all other parameters maintained at default values. The highest-scoring docked conformation was selected as the binding conformation. The mutant T-2 toxin complex structures were obtained directly from the wild-type T-2 toxin complex structures via point mutations introduced in PyMOL, followed by kinetic simulation optimization. These complexes were then used for subsequent molecular dynamics simulations.

### 5.3. Construction of Site-Directed Mutagenesis

Taking the previously constructed recombinant plasmid pET-28a-akr13b3 as a template, the corresponding primers were synthesized according to Table S1, and mutations were introduced via the whole-plasmid PCR method [49]. The 25 μL reaction system contained 10 ng of template (pET-28a-akr13b3), 2 μL each of upstream and downstream primers (10 μM), 2 × Phanta Master Mix, and ddH2O. PCR cycling conditions were: initial denaturation at 98 °C for 30 s, followed by 18 cycles of denaturation at 98 °C for 10 s, annealing at 50 for 10 s, and extension at 72 °C for 30 s, and final complete extension at 72 °C for 3 min. The PCR products were digested with 5 μL of *Dpn I* for 1 h. After recombination, the reaction mixture was directly transformed into *E. coli* BL21 (DE3) via heat shock, spread onto kanamycin-containing resistance plates, and incubated at 37 °C for 12–16 h. Positive transformants with correct sequencing results were selected for subsequent induction and expression.

### 5.4. Screening for Mutants with Enhanced Catalytic Activity

The overnight activated seed culture was inoculated at 1% (*v*/*v*) into 100 mL of LB liquid medium containing kanamycin, and cultured with shaking at 37 °C and 180 r/min. When the OD_600_ of the bacterial culture reached 0.8, IPTG was added, followed by induction culture at 16 °C for 16–20 h. Bacterial pellets were collected after centrifugation at 8000 r/min for 4 min. Under ice bath conditions, the bacterial cell resuspension was ultrasonicated. The lysate was centrifuged at 9000 r/min at low temperature for 20 min, and the supernatant was collected as the crude enzyme solution.

### 5.5. Purification and SDS-PAGE Analysis of Mutants

The supernatant was purified by affinity chromatography due to the fusion of histidine tag in the target protein. The steps for purifying wild-type and mutant enzymes using a crude HisTrap FF affinity column (nickel column) are as follows: after equilibrating the column, add the crude enzyme solution (containing 5 mmol/L imidazole at final concentration) in three fractions using 7–8 column volumes of buffer (50 mmol/L Hepes, 200 mmol/L NaCl, 50 mmol/L imidazole, pH 7.0) to remove contaminating proteins. The target protein is eluted with buffer (50 mmol/L Hepes, 200 mmol/L NaCl, 100 mmol/L imidazole, pH 7.0). SDS-PAGE (sodium dodecyl sulfate polyacrylamide gel electrophoresis) was conducted with separating gel (12%, *w*/*v*) and polyacrylamide stacking gel (5%, *w*/*v*), which was used to analyze the purity, integrity, and molecular mass weight of wild type and mutants.

### 5.6. Protein Determination and Enzyme Activity Assay

The reaction was conducted at 37 °C in 50 mM Tris-HCl buffer (pH 7.5) containing 100 μg of enzyme, 50μM T-2 toxin, and 500μM NADPH in a total volume of 200 μL. The mixture was incubated for 8 h under the same conditions. Enzyme activity was measured using high-performance liquid chromatography. Changes in T-2 toxin content were detected at a UV wavelength of 208 nm. The mobile phase is methanol/water in a 7:3 ratio. Under these conditions, the T-2 toxin standard exhibited a linear response over the tested concentration range (Figure S7). Enzyme activity was defined as follows: enzyme activity was defined under standard conditions (37 °C, pH 7.0), where one unit (U) degrades 1 μg of T-2 toxin per minute. Specific activity is reported as units per milligram of pure enzyme (U/mg).

### 5.7. Enzymatic Characterization of Wild Type and Its Mutant and Enzyme Kinetics Measurements

The characteristics of the wild-type enzyme and its mutants were evaluated by analyzing several key indicators: the optimal temperature for the enzymatic reaction, thermal stability across at a specific temperature, the optimal pH for the enzymatic reaction, pH stability and the kinetic parameters. For the determination of optimum temperature, the enzyme activities were measured at various temperatures (from 25 to 60 °C with a temperature interval of 5 °C) using the assay described in the section on “Protein Determination and Enzyme Activity Assay”. The maximum enzyme activity was defined as 100%, with activities at all other temperatures calculated as percentages relative to this maximum. The thermostability of the enzymes at 55 °C was assessed by incubating them at this temperature and measuring the residual activity at regular time intervals. Enzyme activity was measured in buffers of pH 4.0–10.0 to determine the optimum pH for the enzyme reaction. The enzyme solution was incubated overnight at 4 °C under different pH conditions (4.0–10.0), after which its residual enzymatic activity was measured to determine the pH stability of the enzyme.

The kinetic parameters (*K_m_*, *k_cat_*, and *k_cat_*/*K_m_*) of purified AKR13B3 and its optimum mutant were calculated using non-linear regression. The enzyme activity of AKR13B3 and its optimum mutant were measured by using T-2 (substrate) in the concentration range of 100–500 μM at optimum temperature and pH 7.

### 5.8. LC–MS Analysis of Degradation Products of T-2 Toxin

The procedure was as follows: prepare the reaction system of mutant R134F/D217A with T-2 toxin, set up a blank control without adding mutant R134F/D217A(CK), and react for 24 h. The samples were filtered through a 0.22 µm membrane filter and then directly injected into the LC–MS system for analysis [50]. Analysis was performed using ultra-high performance liquid chromatography-quadrupole time-of-flight mass spectrometry (UHPLC-QTOF, Agilent, US, 1290 Infinity II-6546, Agilent Technologies, Santa Clara, CA, USA). Chromatographic separation was carried out on an Eclipse Plus C18 chromatographic column (50 mm × 2.1 mm, 1.8 µm) and maintained at 30 °C. The mobile phase is a 0.1% formic acid aqueous solution (Solvent A) and methanol (solvent B). The gradient elution program was adopted: starting from 95% A to 5% A, with a flow rate of 0.3 mL/min, and the total running time was 17 min. The injection volume is 2.0 µL. Mass spectrometry detection operates using an electrospray ionization (ESI) source in a positive ion mode. The key gas source parameters are set as follows: sheath gas flow rate: 11 mL/min; auxiliary gas flow rate: 11 mL/min; spray voltage: 3500 V; capillary temperature: 350 °C. Full-scan MS data within the mass range of 50–2000 *m*/*z* were obtained. All solvents, including formic acid and methanol, and ultrapure water were of HPLC grade.

### 5.9. Three-Dimensional Structural Modeling and Molecular Dynamics Simulations

Whole-atom molecular dynamics simulations were conducted on the small molecule-protein complexes derived from docking as initial structures, with the utilization of AMBER 24 software [51]. Prior to the commencement of simulations, the charges of the small molecules were computed via the antechamber module and Gaussian 09 (Revision E.01) software, employing Hartree–Fock (HF) SCF/6–31G* calculations [52,53]. Subsequently, the small molecule and protein were subjected to modeling using the GAFF2 small-molecule force field and the ff14SB protein force field, respectively [54,55]. Hydrogen atoms were incorporated into each system through the LEaP module. The system was solvated in a truncated octahedral TIP3P water box with a 10 Å buffer between the solute and the box boundaries [56]. Na+/Cl− ions were introduced into the system to achieve charge neutralization. Ultimately, the topology and parameter files needed for the simulation were generated.

First, energy minimization was performed on the system, including 2500 steps of the steepest descent method and 2500 steps of the conjugate gradient method. After the system energy minimization was completed, the system was heated for 200 ps at a constant volume and constant heating rate, allowing the system temperature to slowly rise from 0 K to 298.15 K. With the system temperature maintained at 298.15 K, a 500 ps NVT (isothermal–isochoric) ensemble simulation was conducted to ensure the solvent molecules were further uniformly distributed in the solvent box. Subsequently, a 500 ps equilibrium simulation of the entire system was carried out under NPT (isothermal–isobaric) conditions. Finally, a 100 ns NPT (isothermal–isobaric) ensemble simulation of the complex system was performed under periodic boundary conditions. During the simulation, the cutoff distance for non-bonded interactions was set to 10 Å; the Particle Mesh Ewald (PME) method was used to calculate long-range electrostatic interactions [57], the SHAKE method was employed to constrain the bond lengths of hydrogen atoms [58], and the Langevin algorithm was applied for temperature control [59] with collision frequency (γ) set to 2 ps^−1^. The system pressure was 1 atm, the integration time step was 2 fs, and the trajectory was saved every 10 ps for subsequent analysis.

The binding free energy between the protein and ligand in all systems was calculated using the MM/GBSA method [30,31]. For these calculations, the MD trajectory from 90 to 100 ns was used.

### 5.10. Statistical Analysis

Each assay was performed in triplicate, and the results are shown as the means of three repetitions ± standard deviation. All statistical analyses were evaluated by using GraphPad Prism 8.0.

## Figures and Tables

**Table 1:** **Specific activities and kinetic parameters of WT and its mutant enzymes.**

Enzyme	Enzyme Activities (U·ml^−1^)	Specific Activities (U·mg^−1^)	*K_m_* (μM)	*k_cat_* (s^−1^)	*k_cat_*/*K_m_* (mM^−1^·s^−1^)
WT	40.31 ± 1.77 ^d^	80.10 ± 1.35 ^d^	286.30 ± 15.01 ^a^	0.66 ± 0.003 ^d^	2.31
R134F	93.60 ± 2.45 ^b^	189.30 ± 1.71 ^b^	211.56 ± 8.12 ^c^	1.01 ± 0.001 ^b^	4.77
D217A	74.50 ± 1.83 ^c^	148.98 ± 1.12 ^c^	247.58 ± 4.59 ^b^	0.77 ± 0.001 ^c^	3.11
R134F/D217A	108.72 ± 2.42 ^a^	217.40 ± 2.81 ^a^	163.61 ± 7.12 ^d^	1.19 ± 0.003 ^a^	7.27

Note: Different lowercase letters in the same column indicate significant difference at 0.05 level.

**Table 2:** **Binding energy of wild type and R134F/D217A.**

Energy (kcal/mol)	System Name	
	WT	R134F/D217A
**Δ*E*vdw**	−23.94 ± 1.75	−33.79 ± 2.20
**Δ*E*elec**	−1.76 ± 1.14	−10.50 ± 2.72
**ΔGGB**	12.17 ± 0.86	28.11 ± 3.04
**ΔGSA**	−3.03 ± 0.23	−5.13 ± 0.32
**ΔGbind**	−16.57 ± 1.43	−21.31 ± 2.50

## Data Availability

The data presented in this study are available on request from the corresponding author, who, as the project manager, controls access to the data in order to maintain the integrity of the incomplete research project.

## Supplementary Materials

The following supporting information can be downloaded at: https://www.mdpi.com/article/10.3390/toxins18040158/s1, Figure S1: The chemical structures of the three toxins; Figure S2: The three-dimensional structure of the complex formed between AKR13B3 and its cofactor NADPH was predicted using AlphaFold 3; Figure S3: The substrates 3-keto-DON and T-2 toxin were docked against the AKR13B3 structure by semi-rigid docking with AutoDockTools-1.2.3; Figure S4: SDS-PAGE analysis of purified wild type and mutant enzymes; Figure S5: Degradation rate of 3-keto-DON by the mutant; Figure S6: LC–MS analysis of T-2 toxin degradation by the R134F/D217A mutant; Figure S7: Standard curve for the quantification of T-2 toxin by HPLC-UV; Figure S8: Plasmid Map of AKR13B3; Table S1: Oligonucleotide primers used in mutation; Table S2: Relative enzyme activity of mutants.

## References

[1] Taheur F.B., Kouidhi B., Al Qurashi Y.M.A., Salah-Abbès J.B., Chaieb K. (2019). Biotechnology of mycotoxins detoxification using microorganisms and enzymes. Toxicon.

[2] Li Y., Wang Z., Beier R.C., Shen J., De Smet D., De Saeger S., Zhang S. (2011). T-2 toxin, a trichothecene mycotoxin: Review of toxicity, metabolism, and analytical methods. J. Agric. Food Chem..

[3] Yang J., Zhu Y., Han Y., Ke H., Zhang J., Wang M.-W., Lei X. (2025). Developing Fhb7-Derived Enzymes with High Thermostability for Detoxification of T-2 Toxin through Ancestral Sequence Reconstruction. ACS Catal..

[4] Liu M., Zhao L., Wei J.-T., Huang Y.-X., Khalil M.M., Wu W.-D., Kuča K., Sun L.-H. (2023). T-2 toxin-induced intestinal damage with dysregulation of metabolism, redox homeostasis, inflammation, and apoptosis in chicks. Arch. Toxicol..

[5] Zhang J., Li H., Zhang E., Lu Y., Liu B., Yan K., Yang X., Lv H. (2025). Trichothecenes toxicity in humans and animals: Unraveling the mechanisms and harnessing phytochemicals for prevention. Comp. Biochem. Physiol. C Toxicol. Pharmacol..

[6] Yao J., Ouyang B., Xu W., Xie Y., Mu W. (2025). An overview of the physical, chemical and biological strategies for the removal of emerging mycotoxins: Recent advances and future perspectives. Food Control.

[7] Cheng G., Liu C., Wang X., Ma H., Pan Y., Huang L., Hao H., Dai M., Yuan Z. (2014). Structure-function analysis of porcine cytochrome P450 3A29 in the hydroxylation of T-2 toxin as revealed by docking and mutagenesis studies. PLoS ONE.

[8] Chlebicz A., Śliżewska K. (2020). In Vitro Detoxification of Aflatoxin B(1), Deoxynivalenol, Fumonisins, T-2 Toxin and Zearalenone by Probiotic Bacteria from Genus Lactobacillus and Saccharomyces cerevisiae Yeast. Probiotics Antimicrob. Proteins.

[9] Nguyen T., Chen X., Ma L., Feng Y. (2024). Mycotoxin Biodegradation by Bacillus Bacteria—A Review. Toxins.

[10] Garai E., Risa A., Varga E., Cserháti M., Kriszt B., Urbányi B., Csenki Z. (2020). Qualifying the T-2 Toxin-Degrading Properties of Seven Microbes with Zebrafish Embryo Microinjection Method. Toxins.

[11] Fang J., Sheng L., Ye Y., Ji J., Sun J., Zhang Y., Sun X. (2025). Recent advances in biosynthesis of mycotoxin-degrading enzymes and their applications in food and feed. Crit. Rev. Food Sci. Nutr..

[12] Niu J., Ma B., Shen J., Chi H., Zhou H., Lu Z., Lu F., Zhu P. (2023). Structure-guided steric hindrance engineering of Devosia strain A6–243 quinone-dependent dehydrogenase to enhance its catalytic efficiency. J. Agric. Food Chem..

[13] Farhan M., Hasani I.W., Khafaga D.S., Ragab W.M., Ahmed Kazi R.N., Aatif M., Muteeb G., Fahim Y.A. (2025). Enzymes as Catalysts in Industrial Biocatalysis: Advances in Engineering, Applications, and Sustainable Integration. Catalysts.

[14] Lutz S., Iamurri S.M. (2018). Protein Engineering: Past, Present, and Future. Methods Mol. Biol..

[15] MacDonald J.T., Freemont P.S. (2016). Computational protein design with backbone plasticity. Biochem. Soc. Trans..

[16] Castillo-Orellana C., Vöhringer-Martinez E. (2025). Fast Rational Enzyme Design by Computational Non-Equilibrium Alchemical Transformations. Chem. Commun..

[17] Sharma A., Gupta G., Ahmad T., Mansoor S., Kaur B. (2021). Enzyme engineering: Current trends and future perspectives. Food Rev. Int..

[18] Acebes S., Fernandez-Fueyo E., Monza E., Lucas M.F., Almendral D., Ruiz-Dueñas F.J., Lund H., Martinez A.T., Guallar V. (2016). Rational enzyme engineering through biophysical and biochemical modeling. ACS Catal..

[19] He J.W., Bondy G.S., Zhou T., Caldwell D., Boland G.J., Scott P.M. (2015). Toxicology of 3-epi-deoxynivalenol, a deoxynivalenol-transformation product by Devosia mutans 17-2-E-8. Food Chem. Toxicol..

[20] Luo X., Wang Y.-J., Shen W., Zheng Y.-G. (2016). Activity improvement of a Kluyveromyces lactis aldo-keto reductase KlAKR via rational design. J. Biotechnol..

[21] Wang Y., Wang G., Dai Y., Wang Y., Lee Y.-W., Shi J., Xu J. (2020). Biodegradation of deoxynivalenol by a novel microbial consortium. Front. Microbiol..

[22] Yang X., Zhu H., Shi L., Song T., Gong W., He S., Shan S., Xu C., Zhou Z. (2025). AlphaFold-guided structural analyses of nucleosome binding proteins. Nucleic Acids Res..

[23] Gong X., Zhou H., Huang Q. (2025). Assessing AlphaFold 3 for Per-and Polyfluoroalkyl Substances Docking in Protein Structures. Environ. Sci. Technol..

[24] Ravindranath P.A., Forli S., Goodsell D.S., Olson A.J., Sanner M.F. (2015). AutoDockFR: Advances in protein-ligand docking with explicitly specified binding site flexibility. PLoS Comput. Biol..

[25] Laskowski R.A., Swindells M.B. (2011). LigPlot+: Multiple ligand-protein interaction diagrams for drug discovery. J. Chem. Inf. Model..

[26] Yang S., De Boevre M., Zhang H., De Ruyck K., Sun F., Zhang J., Jin Y., Li Y., Wang Z., Zhang S. (2017). Metabolism of T-2 toxin in farm animals and human in vitro and in chickens in vivo using ultra high-performance liquid chromatography-quadrupole/time-of-flight hybrid mass spectrometry along with online hydrogen/deuterium exchange technique. J. Agric. Food Chem..

[27] Wang R., Wang S., Xu Y., Yu X. (2020). Enhancing the thermostability of Rhizopus chinensis lipase by rational design and MD simulations. Int. J. Biol. Macromol..

[28] Zheng F., Tu T., Wang X., Wang Y., Ma R., Su X., Xie X., Yao B., Luo H. (2018). Enhancing the catalytic activity of a novel GH5 cellulase Gt Cel5 from Gloeophyllum trabeum CBS 900.73 by site-directed mutagenesis on loop 6. Biotechnol. Biofuels.

[29] Sarmiento-Pavía P.D., Sosa-Torres M.E. (2021). Bioinorganic insights of the PQQ-dependent alcohol dehydrogenases. JBIC J. Biol. Inorg. Chem..

[30] Hou T., Wang J., Li Y., Wang W. (2011). Assessing the performance of the MM/PBSA and MM/GBSA methods. 1. The accuracy of binding free energy calculations based on molecular dynamics simulations. J. Chem. Inf. Model..

[31] Genheden S., Ryde U. (2015). The MM/PBSA and MM/GBSA methods to estimate ligand-binding affinities. Expert Opin. Drug Discov..

[32] Liu Y., Xu G., Zhou J., Ni J., Zhang L., Hou X., Yin D., Rao Y., Zhao Y.-L., Ni Y. (2020). Structure-guided engineering of D-carbamoylase reveals a key loop at substrate entrance tunnel. ACS Catal..

[33] Yin C., Zheng T., Chang X. (2017). Biosynthesis of S-Adenosylmethionine by Magnetically Immobilized Escherichia coli Cells Highly Expressing a Methionine Adenosyltransferase Variant. Molecules.

[34] Zhou Y., Jiao L., Shen J., Chi H., Lu Z., Liu H., Lu F., Zhu P. (2022). Enhancing the Catalytic Activity of Type II L-Asparaginase from Bacillus licheniformis through Semi-Rational Design. Int. J. Mol. Sci..

[35] Koch U., Biasiol G., Brunetti M., Fattori D., Pallaoro M., Steinkühler C. (2001). Role of charged residues in the catalytic mechanism of hepatitis C virus NS3 protease: Electrostatic precollision guidance and transition-state stabilization. Biochemistry.

[36] Cao T.P., Yi H., Dhanasingh I., Ghosh S., Choi J.M., Lee K.H., Ryu S., Kim H.S., Lee S.H. (2020). Non-catalytic-Region Mutations Conferring Transition of Class A β-Lactamases Into ESBLs. Front. Mol. Biosci..

[37] Freire L., Sant’Ana A.S. (2018). Modified mycotoxins: An updated review on their formation, detection, occurrence, and toxic effects. Food Chem. Toxicol..

[38] Wang J., Sufar E.K., Bernhoft A., Seal C., Rempelos L., Hasanaliyeva G., Zhao B., Iversen P.O., Baranski M., Volakakis N. (2024). Mycotoxin contamination in organic and conventional cereal grain and products: A systematic literature review and meta-analysis. Compr. Rev. Food Sci. Food Saf..

[39] Wang Y., Chen Y., Jiang L., Huang H. (2022). Improvement of the enzymatic detoxification activity towards mycotoxins through structure-based engineering. Biotechnol. Adv..

[40] Song Z., Zhang Q., Wu W., Pu Z., Yu H. (2023). Rational design of enzyme activity and enantioselectivity. Front. Bioeng. Biotechnol..

[41] Zhang Z., Li Z., Yang M., Zhao F., Han S. (2024). Machine learning-guided multi-site combinatorial mutagenesis enhances the thermostability of pectin lyase. Int. J. Biol. Macromol..

[42] Osuna S., Jimenez-Oses G., Noey E.L., Houk K. (2015). Molecular dynamics explorations of active site structure in designed and evolved enzymes. Acc. Chem. Res..

[43] Wang F., Ma X., Sun Y., Guo E., Shi C., Yuan Z., Li Y., Li Q., Lu F., Liu Y. (2023). Structure-guided engineering of a protease to improve its activity under cold conditions. J. Agric. Food Chem..

[44] Zou S.-P., Zheng Y.-G., Wu Q., Wang Z.-C., Xue Y.-P., Liu Z.-Q. (2018). Enhanced catalytic efficiency and enantioselectivity of epoxide hydrolase from Agrobacterium radiobacter AD1 by iterative saturation mutagenesis for (R)-epichlorohydrin synthesis. Appl. Microbiol. Biotechnol..

[45] Wang Y., Wang Z., Yu H., Teng H., Wu J., Xu J., Yang L. (2024). Enhancing the Thermostability and Catalytic Activity of the Lipase from Rhizopus oryzae via Introducing N-Glycosylation. J. Agric. Food Chem..

[46] Gao H., Niu J., Yang H., Lu Z., Zhou L., Meng F., Lu F., Chen M. (2021). Epimerization of deoxynivalenol by the Devosia strain A6-243 assisted by pyrroloquinoline quinone. Toxins.

[47] DeLano W.L. (2002). Pymol: An open-source molecular graphics tool. CCP4 Newsl. Protein Crystallogr..

[48] Eberhardt J., Santos-Martins D., Tillack A.F., Forli S. (2021). AutoDock Vina 1.2.0: New Docking Methods, Expanded Force Field, and Python Bindings. J. Chem. Inf. Model..

[49] Sambrook J., Russell D.W. (2006). The inoue method for preparation and transformation of competent *E. coli*: “Ultra-competent” cells. Cold Spring Harb. Protoc..

[50] Maria W., Tanja W., Florian H.b., Gerald S., Michael G., Hans-Ulrich H. (2012). Identification and Apoptotic Potential of T-2 Toxin Metabolites in Human Cells. J. Agric. Food Chem..

[51] Salomon-Ferrer R., Case D.A., Walker R.C. (2013). An overview of the Amber biomolecular simulation package. WIREs Comput. Mol. Sci..

[52] Frisch M., Trucks G., Schlegel H., Scuseria G., Robb M., Cheeseman J., Scalmani G., Barone V., Mennucci B., Petersson G. (2009). Gaussian 09.

[53] Wang J., Wang W., Kollman P.A., Case D.A. (2001). Antechamber: An accessory software package for molecular mechanical calculations. J. Am. Chem. Soc..

[54] Wang J., Wolf R.M., Caldwell J.W., Kollman P.A., Case D.A. (2004). Development and testing of a general amber force field. J. Comput. Chem..

[55] Maier J.A., Martinez C., Kasavajhala K., Wickstrom L., Hauser K.E., Simmerling C. (2015). ff14SB: Improving the accuracy of protein side chain and backbone parameters from ff99SB. J. Chem. Theory Comput..

[56] Mark P., Nilsson L. (2001). Structure and dynamics of the TIP3P, SPC, and SPC/E water models at 298 K. J. Phys. Chem. A.

[57] Sagui C., Darden T.A. (1999). Molecular dynamics simulations of biomolecules: Long-range electrostatic effects. Annu. Rev. Biophys..

[58] Kräutler V., Van Gunsteren W.F., Hünenberger P.H. (2001). A fast SHAKE algorithm to solve distance constraint equations for small molecules in molecular dynamics simulations. J. Comput. Chem..

[59] Larini L., Mannella R., Leporini D. (2007). Langevin stabilization of molecular-dynamics simulations of polymers by means of quasisymplectic algorithms. J. Chem. Phys..

