# Supplementary material for: Structure-Based Design and Mechanistic Insight for Enhanced Catalytic Activity of Aldo/Keto Reductase AKR13B3 from Devosia A6-243 Toward T-2 Toxin
Source: Toxins (Basel). 2026 Mar 26;18(4):158. doi: 10.3390/toxins18040158 (PMC13120364; doi:10.3390/toxins18040158)
Supplement: Supplementary file 1 [file toxins-18-00158-s001.zip › toxins-4135811-supplementary.pdf]

Supplementary Materials: Structure-Based Design and Mechanistic Insight for Enhanced Catalytic Activity of Aldo/Keto Reductase AKR13B3 from *Devosia* A6-243 Toward T-2 Toxin

Jiali Liu, Huibing Chi, Xiaoyu Zhu, Qingwei Jiang, Zhaoxin Lu, Ping Zhu and Fengxia Lu

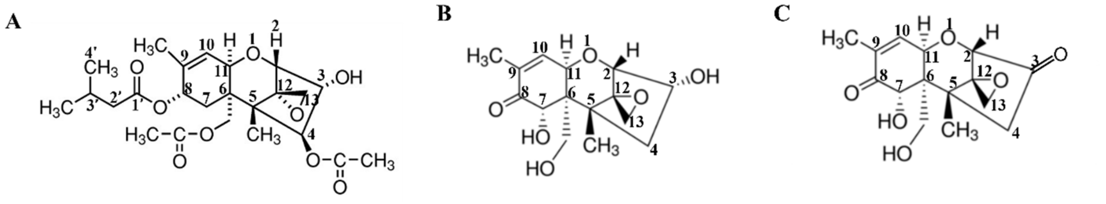

Figure S1. The chemical structures of the three toxins. (A), (B), and (C) are the chemical structural formulas of T-2 toxin, DON, and 3-keto-DON, respectively.

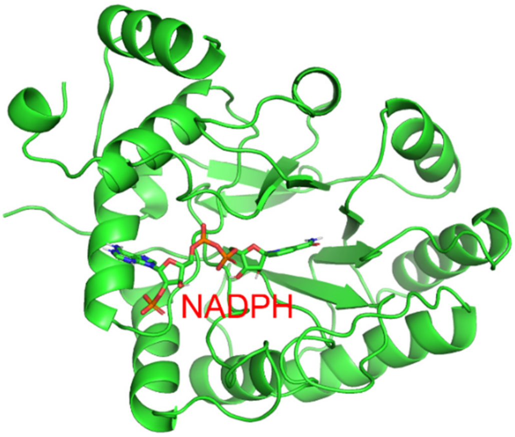

Figure S2. The three-dimensional structure of the complex formed between AKR13B3 and its cofactor NADPH was predicted using AlphaFold 3. Green small sticks represent NADPH.

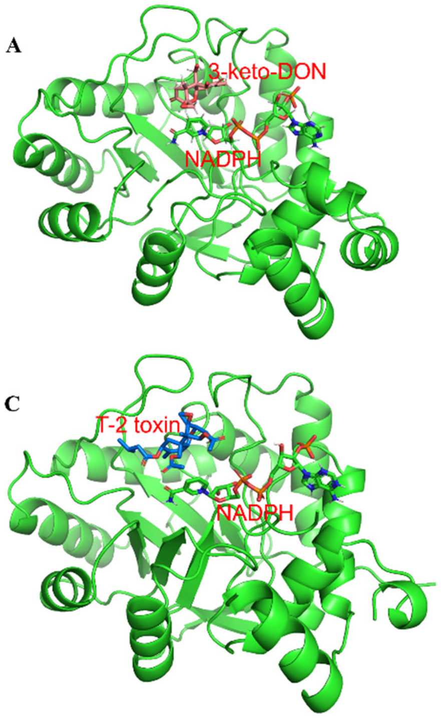

Figure S3. The substrates 3-keto-DON and T-2 toxin were docked against the AKR13B3 structure by semi-rigid docking with AutoDockTools-1.2.3. (A) docking model with 3-keto-DON as the substrate. Pink sticks represent the small molecule, green sticks denote NADPH, green cartoons indicate the protein. (B) (C) (D) three distinct docking models for the substrate T-2 toxin. Blue sticks represent the small molecule, green sticks denote NADPH, green cartoons indicate the protein.

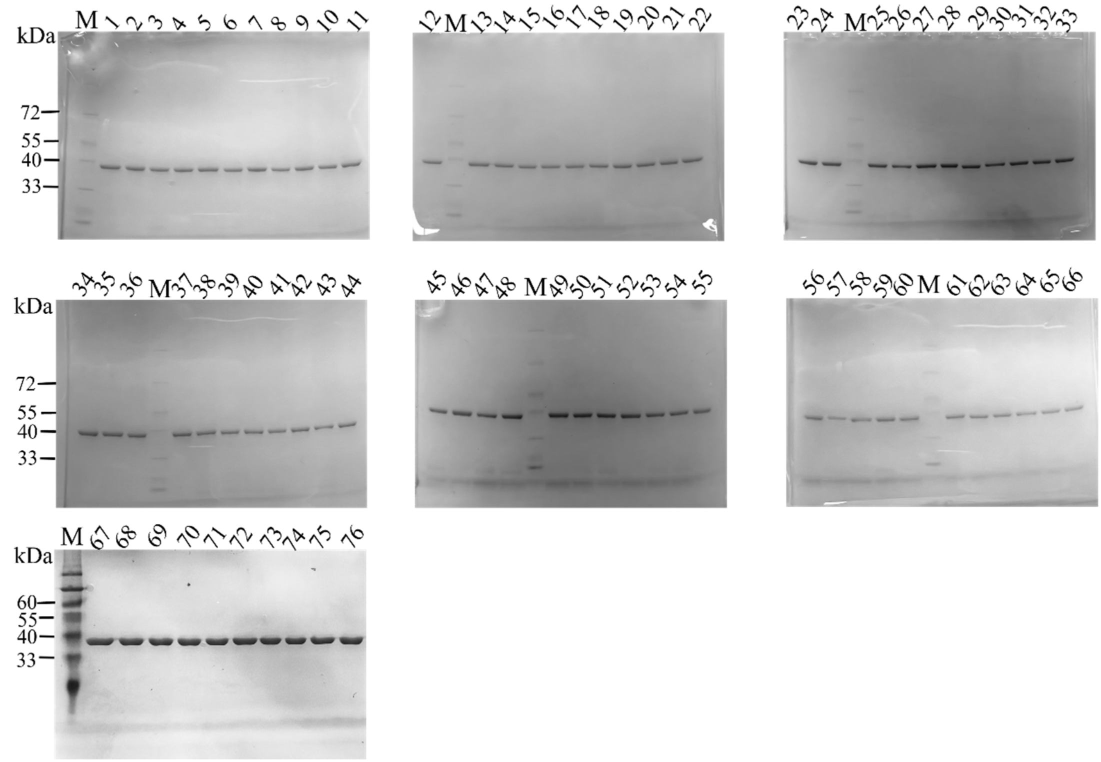

Figure S4. SDS-PAGE analysis of purified wild type and mutant enzymes. M. Standard protein marker; Lanes 1 through 76 are as follows:A214R, A214C, A214D, A214E, A214F, A214G,A214H, A214I,A214K,A214L, A214M, A214N, A214P, A214Q, A214T, A214V, A214W, A214Y, A214S, D217R, D217C, D217A, D217E, D217F, D217G, D217H, D217I, D217K, D217L, D217M, D217N, D217P, D217Q, D217T, D217V, D217W, D217Y, D217S, R134D, R134C, R134A, R134EF, R134G, R134H, R134I, R134K, R134L, R134M, R134N, R134P, R134Q, R134T, R134V, R134W, R134Y, R134S, W102R, W102C, W102A, W102E, W102F, W102G, W102H, W102I, W102K, W102L, W102M, W102N, W102P, W102Q, W102T, W102V, W102D, W102Y, W102S.

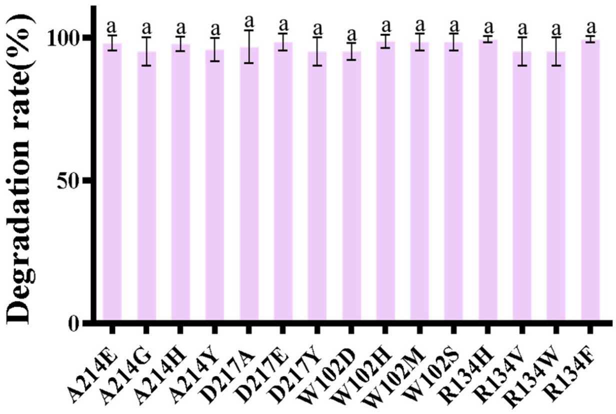

Figure S5. Degradation rate of 3-keto-DON by the mutant. Note: different lowercase letters in histogram indicate that there is significant difference at 0.05 level. The same applies to the following.

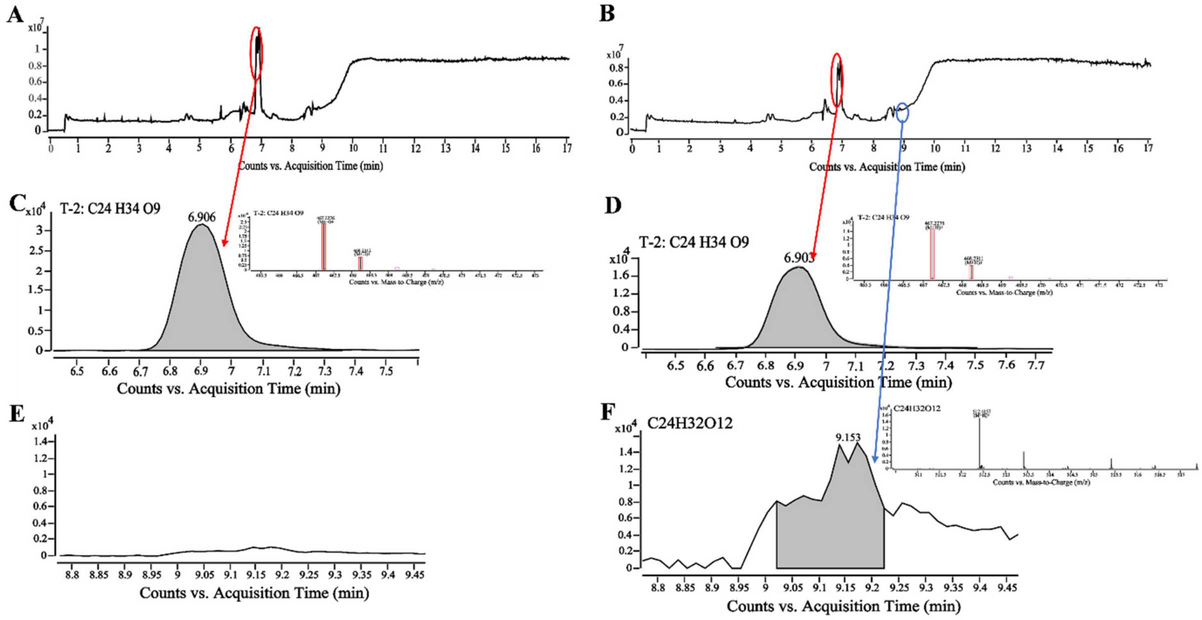

Figure S6. LC–MS analysis of T-2 toxin degradation by the R134F/D217A mutant. (A) The total ion chromatogram (TIC) of the reaction system containing T-2 toxin but without the addition of the R134F/D217A mutant. (B) Total ion chromatogram (TIC) of the reaction system containing T-2 toxin and the R134F/D217A mutant. (C) T-2 toxin EIC (Extracted Ion Chromatogram) in the blank control without mutant. (Corresponding MS^1 spectrum shown in the top-right inset.). (D) EIC of T-2 toxin in the reaction system containing the R134F/D217A mutant. (The MS^1 spectrum is displayed in the top-right inset.). (E) EIC (Extracted Ion Chromatogram) of the blank control without mutant at approximately 9 minutes. (F) The EIC (Extracted Ion Chromatogram) of product $C_{24}H_{32}O_{12}$ from the reaction system containing the R134F/D217A mutant. The corresponding MS^1 spectrum is provided in the inset at the top right.

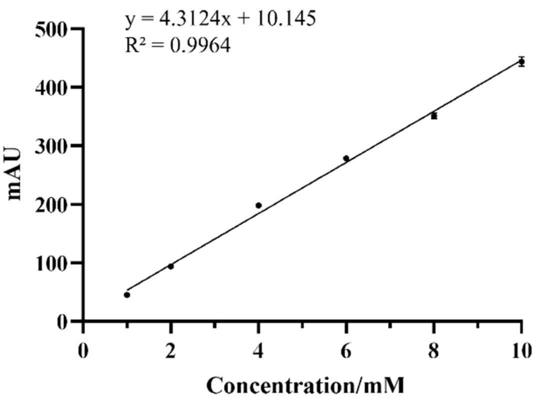

Figure S7. Standard curve for the quantification of T-2 toxin by HPLC-UV.

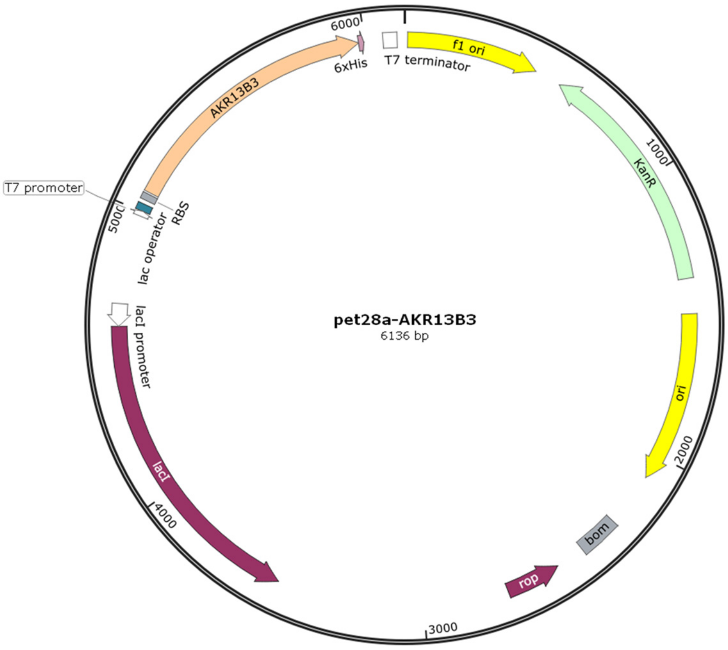

Figure S8. Plasmid Map of AKR13B3.

Table S1. Oligonucleotide primers used in mutation.

Number	Sequence (5′→3′)
A214G-F	ctggttcccgctgGGTggcggtgatctggt
A214G-R	accagatcaccgccACCcagcgggaaccag
A214V-F	ctggttcccgctgGTTggcggtgatctggt
A214V-R	accagatcaccgccAACcagcgggaaccag
A214L-F	ctggttcccgctgCTGggcggtgatctggt
A214L-R	accagatcaccgccCAGcagcgggaaccag
A214I-F	ctggttcccgctgATTggcggtgatctggt
A214I-R	accagatcaccgccAATcagcgggaaccag
A214P-F	ctggttcccgctgCCGggcggtgatctggt
A214P-R	accagatcaccgccCGGcagcgggaaccag
A214F-F	ctggttcccgctgTTTggcggtgatctggt
A214F-R	accagatcaccgccAAAcagcgggaaccag
A214Y-F	ctggttcccgctgTATggcggtgatctggt
A214Y-R	accagatcaccgccATAcagcgggaaccag
A214W-F	ctggttcccgctgTGGggcggtgatctggt
A214W-R	accagatcaccgccCCAcagcgggaaccag
A214S-F	ctggttcccgctgAGCggcggtgatctggt
A214S-R	accagatcaccgccGCTcagcgggaaccag
A214T-F	ctggttcccgctgACCggcggtgatctggt
A214T-R	accagatcaccgccGGTcagcgggaaccag
A214C-F	ctggttcccgctgTGTggcggtgatctggt
A214C-R	accagatcaccgccACAcagcgggaaccag
A214M-F	ctggttcccgctgATGggcggtgatctggt
A214M-R	accagatcaccgccCATcagcgggaaccag
A214N-F	ctggttcccgctgAATggcggtgatctggt
A214N-R	accagatcaccgccATTcagcgggaaccag
A214Q-F	ctggttcccgctgCAGggcggtgatctggt
A214Q-R	accagatcaccgccCTGcagcgggaaccag
A214D-F	ctggttcccgctgGATggcggtgatctggt
A214D-R	accagatcaccgccATCcagcgggaaccag
A214E-F	ctggttcccgctgGAAggcggtgatctggt
A214E-R	accagatcaccgccTTCcagcgggaaccag
A214K-F	ctggttcccgctgAAAggcggtgatctggt
A214K-R	accagatcaccgccTTTcagcgggaaccag
A214R-F	ctggttcccgctgCGTggcggtgatctggt
A214R-R	accagatcaccgccACGcagcgggaaccag
A214H-F	ctggttcccgctgCATggcggtgatctggt
A214H-R	accagatcaccgccATGcagcgggaaccag
A214K-F	ctggttcccgctgAAAggcggtgatctggt
A214K-R	accagatcaccgccTTTcagcgggaaccag
A214R-F	ctggttcccgctgCGTggcggtgatctggt
A214R-R	accagatcaccgccACGcagcgggaaccag

Continued table S1. Oligonucleotide primers used in mutation.

Number	Sequence (5′→3′)
A214H-F	ctggttcccgctgCATggcggtgatctggt
A214H-R	accagatcaccgccATGcagcgggaaccag
D217G-F	cgctggccggcggtGGTctggtggagggtc
D217G-R	gaccctccaccagACCaccgccggccagcg
D217A-F	cgctggccggcggtGCActggtggagggtc
D217A-R	gaccctccaccagTGCaccgccggccagcg
D217V-F	cgctggccggcggtGTTctggtggagggtc
D217V-R	gaccctccaccagAACaccgccggccagcg
D217L-F	cgctggccggcggtCTGctggtggagggtc
D217L-R	gaccctccaccagCAGaccgccggccagcg
D217I-F	cgctggccggcggtATTctggtggagggtc
D217I-R	gaccctccaccagAATaccgccggccagcg
D217P-F	cgctggccggcggtCCGctggtggagggtc
D217P-R	gaccctccaccagCGGaccgccggccagcg
D217F-F	cgctggccggcggtTTTctggtggagggtc
D217F-R	gaccctccaccagAAAaccgccggccagcg
D217Y-F	cgctggccggcggtTATctggtggagggtc
D217Y-R	gaccctccaccagATAaccgccggccagcg
D217W-F	cgctggccggcggtTGGctggtggagggtc
D217W-R	gaccctccaccagCCAaccgccggccagcg
D217S-F	cgctggccggcggtAGCctggtggagggtc
D217S-R	gaccctccaccagGCTaccgccggccagcg
D217T-F	cgctggccggcggtACCctggtggagggtc
D217T-R	gaccctccaccagGGTaccgccggccagcg
D217C-F	cgctggccggcggtTGTctggtggagggtc
D217C-R	gaccctccaccagACAaccgccggccagcg
D217M-F	cgctggccggcggtATGctggtggagggtc
D217M-R	gaccctccaccagCATaccgccggccagcg
D217N-F	cgctggccggcggtAATctggtggagggtc
D217N-R	gaccctccaccagATTaccgccggccagcg
D217Q-F	cgctggccggcggtCAGctggtggagggtc
D217Q-R	gaccctccaccagCTGaccgccggccagcg
D217E-F	cgctggccggcggtGAActggtggagggtc
D217E-R	gaccctccaccagTTCaccgccggccagcg
D217K-F	cgctggccggcggtAAActggtggagggtc
D217K-R	gaccctccaccagTTTaccgccggccagcg
D217R-F	cgctggccggcggtCGTctggtggagggtc
D217R-R	gaccctccaccagACGaccgccggccagcg
D217H-F	cgctggccggcggtCATctggtggagggtc
D217H-R	gaccctccaccagATGaccgccggccagcg
R134G-F	tctggcaattgcacGGTatcgacgccaaga
R134G-R	tcttggcgtcgatACCgtgcaattgccaga

Continued table S1. Oligonucleotide primers used in mutation.

Number	Sequence (5′→3′)
R134A-F	tctggcaattgcacGCAatcgacgccaaga
R134A-R	tcttggcgtcgatTGCgtgcaattgccaga
R134V-F	tctggcaattgcacGTTatcgacgccaaga
R134V-R	tcttggcgtcgatAACgtgcaattgccaga
R134L-F	tctggcaattgcacCTGatcgacgccaaga
R134L-R	tcttggcgtcgatCAGgtgcaattgccaga
R134I-F	tctggcaattgcacATTatcgacgccaaga
R134I-R	tcttggcgtcgatAATgtgcaattgccaga
R134P-F	tctggcaattgcacCCGatcgacgccaaga
R134P-R	tcttggcgtcgatCGGgtgcaattgccaga
R134F-F	tctggcaattgcacTTTatcgacgccaaga
R134F-R	tcttggcgtcgatAAAgtgcaattgccaga
R134Y-F	tctggcaattgcacTATatcgacgccaaga
R134Y-R	tcttggcgtcgatATAgtgcaattgccaga
R134W-F	tctggcaattgcacTGGatcgacgccaaga
R134W-R	tcttggcgtcgatCCAgtgcaattgccaga
R134S-F	tctggcaattgcacAGCatcgacgccaaga
R134S-R	tcttggcgtcgatGCTgtgcaattgccaga
R134T-F	tctggcaattgcacACCatcgacgccaaga
R134T-R	tcttggcgtcgatGGTgtgcaattgccaga
R134C-F	tctggcaattgcacTGTatcgacgccaaga
R134C-R	tcttggcgtcgatACAgtgcaattgccaga
R134M-F	tctggcaattgcacATGatcgacgccaaga
R134M-R	tcttggcgtcgatCATgtgcaattgccaga
R134N-F	tctggcaattgcacAATatcgacgccaaga
R134N-R	tcttggcgtcgatATTgtgcaattgccaga
R134Q-F	tctggcaattgcacCAGatcgacgccaaga
R134Q-R	tcttggcgtcgatCTGgtgcaattgccaga
R134D-F	tctggcaattgcacGATatcgacgccaaga
R134D-R	tcttggcgtcgatATCgtgcaattgccaga
R134E-F	tctggcaattgcacGAAatcgacgccaaga
R134E-R	tcttggcgtcgatTTCgtgcaattgccaga
R134K-F	tctggcaattgcacAAAatcgacgccaaga
R134K-R	tcttggcgtcgatTTTgtgcaattgccaga
R134H-F	tctggcaattgcacCATatcgacgccaaga
R134H-R	tcttggcgtcgatATGgtgcaattgccaga
W102G-F	gcggtcccaatcaaGGTccgccgctggggc
W102G-R	gccccagcggcggACCttgattgggaccgc
W102A-F	gcggtcccaatcaaGCAccgccgctggggc
W102A-R	gccccagcggcggTGCttgattgggaccgc
W102V-F	gcggtcccaatcaaGTTccgccgctggggc
W102V-R	gccccagcggcggAACttgattgggaccgc

Continued table S1. Oligonucleotide primers used in mutation.

Number	Sequence (5′→3′)
W102L-F	gcggtcccaatcaaCTGccgccgctggggc
W102L-R	gccccagcggcggCAGttgattgggaccgc
W102I-F	gcggtcccaatcaaATTccgccgctggggc
W102I-R	gccccagcggcggAATttgattgggaccgc
W102P-F	gcggtcccaatcaaCCGccgccgctggggc
W102P-R	gccccagcggcggCGGttgattgggaccgc
W102F-F	gcggtcccaatcaaTTTccgccgctggggc
W102F-R	gccccagcggcggAAAttgattgggaccgc
W102Y-F	gcggtcccaatcaaTATccgccgctggggc
W102Y-R	gccccagcggcggATAttgattgggaccgc
W102S-F	gcggtcccaatcaaAGCccgccgctggggc
W102S-R	gccccagcggcggGCTttgattgggaccgc
W102T-F	gcggtcccaatcaaACCccgccgctggggc
W102T-R	gccccagcggcggGGTttgattgggaccgc
W102C-F	gcggtcccaatcaaTGTccgccgctggggc
W102C-R	gccccagcggcggACAttgattgggaccgc
W102M-F	gcggtcccaatcaaATGccgccgctggggc
W102M-R	gccccagcggcggCATttgattgggaccgc
W102N-F	gcggtcccaatcaaAATccgccgctggggc
W102N-R	gccccagcggcggATTttgattgggaccgc
W102Q-F	gcggtcccaatcaaCAGccgccgctggggc
W102Q-R	gccccagcggcggCTGttgattgggaccgc
W102D-F	gcggtcccaatcaaGATccgccgctggggc
W102D-R	gccccagcggcggATCttgattgggaccgc
W102E-F	gcggtcccaatcaaGAAccgccgctggggc
W102E-R	gccccagcggcggTTCttgattgggaccgc
W102K-F	gcggtcccaatcaaAAAccgccgctggggc
W102K-R	gccccagcggcggTTTttgattgggaccgc
W102R-F	gcggtcccaatcaaCGTccgccgctggggc
W102R-R	gccccagcggcggACGttgattgggaccgc
W102H-F	gcggtcccaatcaaCATccgccgctggggc
W102H-R	gccccagcggcggATGttgattgggaccgc

*Mutation sites are indicated in uppercase letters.

Table S2. Relative enzyme activity of mutants.

	**Relative Activity (%)**
WT	100.66±1.15[d]
R134F	234.19±17.59[b]
D217A	185.90±8.75[c]
R134F/D217A	277.92±3.14[a]

Note: Different lowercase letters in the same column indicate significant difference at 0.05 level
